# Comparison of revision surgery after implant-based breast reconstruction between smooth, textured, and polyurethane-covered implants: results from the Dutch Breast Implant Registry

**DOI:** 10.1093/bjs/znaf082

**Published:** 2025-05-17

**Authors:** J Xavier Harmeling, J Juliët Vrolijk, Erik Heeg, Babette E Becherer, Hinne A Rakhorst, Eveline M L Corten, Marta Fiocco, Marc A M Mureau

**Affiliations:** Department of Plastic and Reconstructive Surgery, Erasmus MC Cancer Institute, University Medical Center Rotterdam, Rotterdam, Zuid-Holland, The Netherlands; Department of Plastic and Reconstructive Surgery, Maastricht University Medical Center +, Maastricht, The Netherlands; Dutch Institute for Clinical Auditing, Leiden, The Netherlands; Department of Plastic, Reconstructive and Hand Surgery, Amsterdam University Medical Center, Amsterdam, The Netherlands; Department of Plastic and Reconstructive Surgery, Erasmus MC Cancer Institute, University Medical Center Rotterdam, Rotterdam, Zuid-Holland, The Netherlands; Allegro Medical, Hilversum, Noord-Holland, The Netherlands; Dutch Institute for Clinical Auditing, Leiden, The Netherlands; Department of Plastic, Reconstructive, and Hand Surgery, Medisch Spectrum Twente, Enschede, The Netherlands; Department of Plastic, Reconstructive, and Hand Surgery, ZGT, Almelo, The Netherlands; Department of Plastic and Reconstructive Surgery, Erasmus MC Cancer Institute, University Medical Center Rotterdam, Rotterdam, Zuid-Holland, The Netherlands; Mathematical Institute, Leiden University, Leiden, The Netherlands; Department of Biomedical Data Science, Section Medical Statistics, Leiden University Medical Center, Leiden, The Netherlands; Princess Máxima Center for Pediatric Oncology, Utrecht, The Netherlands; Department of Plastic and Reconstructive Surgery, Erasmus MC Cancer Institute, University Medical Center Rotterdam, Rotterdam, Zuid-Holland, The Netherlands; Dutch Institute for Clinical Auditing, Leiden, The Netherlands

## Abstract

**Background:**

Implant-based breast reconstruction is the most common technique after mastectomy. Breast implants are categorized by surface type as smooth, textured, or polyurethane-covered, each with specific attributed advantages and complication profiles. High-quality comparative studies are, however, limited. This study compared revision incidence and indications for revision among these implant types.

**Methods:**

A prospective, nationwide cohort from the Dutch Breast Implant Registry was analysed. Permanent implants used between 2017 and 2022 for direct-to-implant or two-stage reconstruction were included. Surface-related revision was the primary outcome. Cumulative incidences were estimated using a competing risk model. Cause-specific hazard ratios (HR_cs_) were calculated using univariable and multivariable models, accounting for implant clustering and confounders. Subgroup analyses examined revisions for specific complications.

**Results:**

Of 3996 implants, 76.9% were textured, 12.4% smooth, and 10.8% polyurethane-covered. At 4 years, the cumulative incidence of revision surgeries did not differ between textured (11.1%; 95% c.i. = 9.9 to 12.5), smooth (13.0%; 95% c.i. = 8.5 to 18.4), and polyurethane-covered (16.1%; 95% c.i. = 12.4 to 20.2) implants. Multivariable analysis found no association between surface type and surface-related revision. Subgroup analysis however revealed that polyurethane-covered implants had increased hazards of revision for capsular contracture (HRcs = 2.49; 95% c.i. = 1.24 to 5.01) and asymmetry (HRcs = 2.31; 95% c.i. = 1.33 to 4.02).

**Conclusion:**

After adjusting for confounders and clustering, surface-related revision in a reconstructive setting did not significantly different among breast implant surface types overall. Polyurethane-covered implants may, however, require more revisions due to capsular contracture and asymmetry.

## Teaser

This study compares revision incidence and indications among smooth, textured, and polyurethane-covered breast implants using data from the Dutch Breast Implant Registry (2017–2022). No significant differences in overall revision risk were observed between surface types, but polyurethane-covered implants had a higher chance of revisions due to capsular contracture and asymmetry. These findings highlight specific risks associated with implant surface types in reconstructive settings.

## Introduction

Breast cancer is the most diagnosed malignancy worldwide, with 2.3 million new patients diagnosed each year^1^. Mastectomy is part of its surgical treatment in approximately 30% of patients with an invasive tumour^2^. In addition, approximately 30% of unilateral breast cancer patients opt for contralateral prophylactic mastectomy and about 40% of women with a genetic predisposition for breast cancer choose prophylactic bilateral mastectomy^3–5^. Quality of life after mastectomy is improved significantly by breast reconstruction^6^. Breast reconstruction can be performed using a breast implant, autologous tissue, or a combination of both^7^. Implant-based breast reconstruction (IBBR) is the most commonly performed reconstructive technique^8^.

Breast implants can be classified based on their surface characteristics, with three primary categories identified: smooth, textured, and polyurethane-covered^9^. For decades, there has been an ongoing discussion about implant surface types and their advantages and disadvantages^10^. Capsular contracture, a common complication of IBBR, and its relation to breast implant surface makes up a substantial part of this discussion. A capsule that develops naturally around every breast implant can contract with the breast becoming hard, disfigured, and painful. Originally, polyurethane-covered implants were introduced as an alternative to smooth implants to reduce the risk of capsular contracture, claiming that the polyurethane structure prevents collagen fibres within the capsule from contracting. Subsequently, textured implants were developed to mimic this property^11^. An additional advantage of these non-smooth surfaced breast implants is their greater tissue adherence, which may reduce malposition and rotation^12,13^. Capsular contracture and associated consequences such as asymmetry, malposition, and breast pain are among the most frequently reported indications for revision surgery after IBBR^14–16^.

Revision surgery reduces the quality of life of patients^17,18^. Presently, a paucity of literature exists comparing different breast implant surface types and their associated risk of revision surgery after IBBR. Previous studies, focusing on capsular contracture, suggest a lower risk of capsular contracture for textured implants than smooth implants^19–23^. Additionally, limited evidence indicates that polyurethane-covered implants have a lower risk of capsular contracture than textured implants^24–32^. However, most studies regarding capsular contracture were retrospective, and the few prospective studies lack an adequate control cohort. Moreover, these studies often suffered from other methodological flaws such as short follow-up, small sample sizes and confounding. Nonetheless, manufacturers centralize their marketing strategy around breast implant surface characteristics, claiming significant advantages, specifically regarding the risk of capsular contracture.

The current study aimed to compare the incidence of surface-related revision and revision indications between smooth, textured, and polyurethane-covered breast implants in a prospective, nationwide, population-based breast reconstruction cohort using real-world data from a breast implant registry.

## Methods

### Study design and population

Data were retrieved from the Dutch Breast Implant Registry (DBIR), a mandatory, nationwide, population-based registry in the Netherlands where the patient, surgery, and implant characteristics of all patients undergoing reconstructive or cosmetic surgery with permanent breast implants or tissue expanders are prospectively registered^33,34^. DBIR started in 2015 and has a national capture rate of 97% (8 academic hospitals, 65 regional hospitals, and 32 private clinics)^33–36^. DBIR supports tracking breast implants between medical institutions. The DBIR has been validated through a systematic data verification process^37^.

For this observational study, all permanent breast implants inserted for immediate direct-to-implant or two-stage IBBR after therapeutic or prophylactic mastectomy between September 2017 and November 2022 were included. The pilot period before September 2017, which prompted a major database update, was excluded to increase data quality. Delayed reconstructions, reconstructions involving additional surgical techniques (fat grafting, autologous flaps, mastopexy), reconstructions that were preceded by any previous breast implant surgery (excluding tissue expander insertion as part of a two-stage reconstruction), or non–silicone-filled implants (a rarity in the Netherlands) were excluded from analysis. Exact definitions of all variables used for analysis can be found in the DBIR Data Dictionary (*Digital Content S1*).

Each implant insertion surgery included was required to have a follow-up of at least 60 days because a substantial number of complications in breast implant surgery occur after 30 days^38,39^. The data set was last updated on 17 March 2023. This study follows the STROCSS guideline and the Declaration of Helsinki. It was approved by the scientific board of DBIR and the Medical Ethical Research Committee of the Erasmus Medical Centre (MEC-2023–0300) and registered at DRKS^40,41^.

### Definitions

The primary outcome was surface-related revision surgery, defined as any reoperation after insertion of the permanent implant, during which the implant was repositioned, replaced, or explanted due to an indication possibly related to the implant surface. All remaining revisions were labelled non–surface-related revisions (*Table S1*). Textured implants can be further classified according to roughness and surface area characteristics into subgroups, for example micro- and macro-textured. Various classification systems have been proposed using different approaches and cut-off values^11,42–45^. Because no consensus exists on a preferred classification system, this study did not differentiate between subgroups of textured implants. Immediate reconstruction was defined as implant insertion at the time of mastectomy, and delayed reconstruction was defined as insertion during a separate procedure after mastectomy. Direct-to-implant IBBR was defined as the immediate insertion of a permanent implant following mastectomy without prior tissue expander insertion. Two-stage IBBR was defined as the insertion of a tissue expander after mastectomy followed by a second operation during which the tissue expander was replaced with a permanent implant. Therapeutic mastectomies were performed for breast cancer. Unplanned tissue expander revision was defined as reoperation during the first stage of a two-stage IBBR. The number of applied Infection Control Measures (ICMs) was categorized based on the nationwide median. It included preoperative/postoperative antibiotics, nipple guards, pocket irrigation, glove change, sleeve/Keller funnel, and drains^46^.

### Statistical analysis

Analyses were performed with implants as units of analysis. Baseline characteristics and revision indications were analysed using descriptive statistics. Implants without any revision at the closure of the data set were right censored. Administrative censoring was performed at 4 years. Time to revision was defined as the time from insertion of the permanent implant to its surgical revision. The cumulative incidence of surface-related revision was estimated using a competing risk model with non–surface-related revision as a competing event^47^. Gray’s test assessed differences between the cause-specific cumulative incidences of revision of each implant surface over the entire follow-up^48^. A significant value of *P* indicates that over follow-up as a whole, there is a significant difference in cumulative incidence of surface-related revision between implant surface types.

To quantify the effect of implant surface type on surface-related revision, univariable and multivariable hazard regression models were used to estimate cause-specific hazard ratios (HR_cs_), taking clustering of implants within patients into account and adjusting for confounding factors that were selected based on existing literature and clinical knowledge. As there were sufficient degrees of freedom (at least 10 events per category in the model), all confounding factors were added to the multivariable model, provided they did not show multicollinearity using a variance inflation factor (VIF) ≥ 3 as the cut-off value^49,50^. Unplanned tissue expander revision and implant shape were excluded as confounders because they correlated with the reconstruction type and implant surface respectively. The proportional-hazards assumption was tested with the scaled Schoenfeld residuals. As the proportional-hazards assumption was violated for the type of reconstruction, BMI, reconstruction indication, plane, and acellular dermal matrix (ADM)/mesh use, a stratified hazard regression was estimated with separate baseline hazards for each stratum.

Because capsular contracture has been a central theme in breast implant-related outcome research, subgroup analyses focusing on revision surgery due to capsular contracture and associated revision indications such as asymmetry, malposition, and breast pain were performed. The method of these subgroup analyses mirrors that of the primary investigation.

In all analyses, two-sided *P* < 0.05 were considered statistically significant. All analyses concerning the competing risk models were performed in the R software environment with the mstate and survival library (version 4.2.1)^51,52^.

## Results

A total of 3150 patients and 3996 permanent breast implants met the eligibility criteria (*Fig. S1*). Of the included implants, 76.9% (*n* = 3072) had a textured surface, 12.4% (*n* = 494) a smooth surface, and 10.8% (*n* = 430) a polyurethane-covered surface. Patients had a mean age of 48.4 years (11.8 s.d.). In the study population, 5.1% (*n* = 203) of implants were inserted in patients classified as ASA III or higher, 9.4% (*n* = 376) in current smokers, and 8.6% (*n* = 345) after previous radiotherapy. A total of 78.8% (*n* = 3148) implants were inserted after mastectomy for breast cancer and 21.2% (*n* = 848) after prophylactic mastectomy. A total of 43.6% (*n* = 1743) implants were used in a direct-to-implant reconstruction and 56.4% (*n* = 2253) in a two-stage reconstruction. In the two-stage reconstruction group, 8.4% (*n* = 336) of implants were inserted after an unplanned tissue expander revision during the first stage. A total of 37.3% (*n* = 1489) of implants were placed in a total submuscular plane. An ADM or mesh was used for 11.0% (*n* = 438) of implants. Round implants were used in 19.5% (*n* = 779) and anatomical implants in 80.4% (*n* = 3212) of all reconstructions. The reconstructions were performed in 77 healthcare institutions with a median volume per institution of 75 post-mastectomy reconstructions per year (interquartile range (i.q.r.) 46–103; range = 1–522). Low institutional volumes were observed in clinics where mainly cosmetic breast augmentations are performed. Baseline characteristics per implant surface group are shown in *Table 1*.

**Table 1:** Baseline characteristics at time of permanent breast implant insertion for post-mastectomy reconstruction

	Total (*n* = 3996)	Textured (*n* = 3072)	Smooth (*n* = 494)	Polyurethane (*n* = 430)
**Age (years), mean(s.d.)**	48.4 (11.8)	48.7 (11.8)	47.1 (11.8)	47.5 (12.0)
** **Missing	7 (0.2)	4 (0.1)	2 (0.4)	1 (0.2)
**ASA classification**				
I	1705 (42.7)	1346 (43.8)	191 (38.7)	168 (39.1)
II	2079 (52.0)	1558 (50.7)	287 (58.1)	234 (54.4)
III+	203 (5.1)	162 (5.3)	14 (2.8)	27 (6.3)
Missing	9 (0.2)	6 (0.2)	2 (0.4)	1 (0.2)
**BMI (kg/m^2^), median (i.q.r.)**	23.7 (21.6–26.5)	23.8 (21.6–26.4)	23.5 (21.8–26.4)	23.5 (21.5–26.7)
Missing	112 (3.8)	106 (3.5)	5 (1.0)	1 (0.2)
**Smoking status**				
Non-smoker	3346 (83.7)	2554 (83.1)	426 (86.2)	366 (85.1)
Smoker	376 (9.4)	296 (9.6)	42 (8.5)	38 (8.8)
Missing	274 (6.9)	222 (7.2)	26 (5.3)	26 (6.0)
**Previous radiotherapy**				
No	3549 (88.8)	2737 (89.1)	429 (86.8)	383 (89.1)
Yes	345 (8.6)	238 (7.7)	61 (12.3)	46 (10.7)
Missing	102 (2.6)	97 (3.2)	4 (0.8)	1 (0.2)
Year of insertion*				
2017	149 (3.7)	139 (4.5)	0 (0.0)	10 (2.3)
2018	646 (16.2)	554 (18.0)	4 (0.8)	88 (20.5)
2019	831 (20.8)	647 (21.1)	104 (21.1)	80 (18.6)
2020	903 (22.6)	665 (21.6)	131 (26.5)	107 (24.9)
2021	835 (20.9)	644 (21.0)	116 (23.5)	75 (17.4)
2022	632 (15.8)	423 (13.8)	139 (28.1)	70 (16.3)
**Institutional volume per year**				
<75th percentile	1991 (49.8)	1564 (50.9)	184 (37.2)	243 (56.5)
≥75th percentile	2005 (50.2)	1508 (49.1)	310 (62.8)	187 (43.5)
**Indication for reconstruction**				
Therapeutic mastectomy	3148 (78.8)	2445 (79.6)	380 (76.9)	323 (75.1)
Prophylactic mastectomy	848 (21.2)	627 (20.4)	114 (23.1)	107 (24.9)
**Laterality**				
Unilateral	2023 (50.6)	1583 (51.5)	242 (49.0)	198 (46.0)
Bilateral	1973 (49.4)	1489 (48.5)	252 (51.0)	232 (54.0)
**Type of reconstruction**				
Direct-to-implant	1743 (43.6)	1351 (44.0)	180 (36.4)	212 (49.3)
Two-stage	2253 (56.4)	1721 (56.0)	314 (63.6)	218 (50.7)
**Unplanned TE revision**				
No	1917 (48.0)	1477 (48.1)	252 (51.0)	188 (43.7)
Yes	336 (8.4)	244 (7.9)	62 (12.6)	30 (7.0)
Not applicable: direct-to-implant	1743 (43.6)	1351 (44.0)	180 (36.4)	212 (49.3)
**Incision site**				
Nipple sparing	1663 (41.6)	1250 (40.7)	203 (41.1)	210 (48.8)
Non-nipple sparing	2126 (53.2)	1658 (54.0)	268 (54.3)	200 (46.5)
Other	126 (3.2)	90 (2.9)	17 (3.4)	19 (4.4)
Missing	81 (2.0)	74 (2.4)	6 (1.2)	1 (0.2)
**Plane**				
Total submuscular plane	1489 (37.3)	1206 (39.3)	164 (33.2)	119 (27.7)
Other	2368 (59.3)	1736 (56.5)	322 (65.2)	310 (72.1)
Missing	139 (3.5)	130 (4.2)	8 (1.6)	1 (0.2)
**Number of applied ICMs during implant insertion**				
<4	775 (19.4)	636 (20.7)	96 (19.4)	43 (10.0)
4	1461 (36.6)	1159 (37.7)	211 (42.7)	91 (21.2)
>4	1758 (44.0)	1275 (41.5)	187 (37.9)	296 (68.8)
Missing	2 (0.1)	2 (0.1)	0 (0.0)	0 (0.0)
**ADM/mesh**				
No	3428 (85.8)	2578 (83.9)	463 (93.7)	387 (90.0)
Yes	438 (11.0)	367 (11.9)	28 (5.7)	43 (10.0)
Missing	130 (3.3)	127 (4.1)	3 (0.6)	0 (0.0)
**Implant shape**				
Round	779 (19.5)	284 (9.2)	484 (98.0)	11 (2.6)
Anatomical	3212 (80.4)	2783 (90.6)	10 (2.0)	419 (97.4)
Missing	5 (0.1)	5 (0.2)	0 (0.0)	0 (0.0)
Follow-up (years), median (i.q.r.)	2.4 (1.3–3.7)	2.5 (1.4–3.9)	1.9 (1.0–3.0)	2.6 (1.3–3.9)

ADM, acellular dermal matrix; ICM, infection control measure; PM, pectoralis major; TE, tissue expander. Values are *n* (%) unless otherwise indicated. *2017 and 2022 were incomplete registration years, because data in 2017 were included from September and in 2022 until November.

### Cumulative incidence of surface-related revision

Of 3996 included implants, 90.4% (*n* = 3613) did not undergo surface-related revision surgery, whereas surface-related revision occurred in 9.3% (*n* = 285) of textured implants, 7.7% (*n* = 38) of smooth implants, and 14.0% (*n* = 60) of polyurethane-covered implants. The median follow-up for the complete cohort was 2.4 years (i.q.r. = 1.3–3.7). Median time to surface-related revision was 0.9 years for the complete cohort (i.q.r. = 0.2–1.7; range = 0.0–5.0), 0.8 years for textured implants (i.q.r. = 0.1–1.8; range = 0.0–5.1), 1.0 years for smooth implants (i.q.r. = 0.3–1.7; range = 0.0–3.7), and 1.1 years for polyurethane-covered implants (i.q.r. = 0.6–1.5; range = 0.0–4.3).

**Fig. 1:**
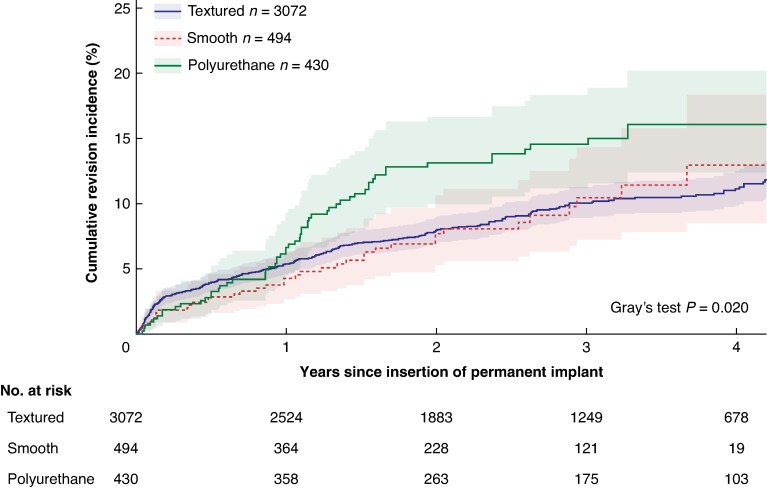
Cumulative incidence of surface-related revision of permanent breast implants inserted for post-mastectomy reconstruction, stratified according to implant surface The curves have not been adjusted for confounders and clustering within patients has not been taken into account. Confidence intervals are displayed by the shadows of the curves.

Over the entire follow-up period, the cumulative incidence of surface-related revision differed significantly between breast implant surface types (*P* = 0.020). At 4 years follow-up, no significant difference in cumulative incidence was observed between textured (11.1%; 95% c.i. = 9.9 to 12.5), smooth (13.0%; 95% c.i. = 8.5 to 18.4), and polyurethane-covered implants (16.1%; 95% c.i. = 12.4 to 20.2) (*Fig. 1*).

The multivariable cause-specific hazard regression found no association between implant surface and surface-related revision (*Table 2*). *Table S2* shows the effect of the selected confounders on surface-related revision. ASA classification III+ (HR_cs_ = 1.91; 95% c.i. = 1.34 to 2.74), smoking (HR_cs_ = 1.52; 95% c.i. = 1.09 to 2.12), and previous radiotherapy (HR_cs_ = 1.50; 95% c.i. = 1.08 to 2.09) were associated with a higher hazard of surface-related revision. Bilateral surgery (HR_cs_ = 0.79; 95% c.i. = 0.63 to 0.99) and direct-to-implant procedures (HR_cs_ = 0.61; 95% c.i. = 0.49 to 0.76) were associated with a lower hazard of surface-related revision.

**Table 2:** Cause-specific hazard regression of surface-related revision of permanent breast implants inserted for post-mastectomy reconstruction

Textured (*n* = 3072, *n* events = 285 (9.3%)) Smooth (*n* = 494, *n* events = 38 (7.7%)) Polyurethane (*n* = 430, *n* events = 60 (14.0%))	HR_cs_ (95% c.i.)
**Unadjusted (univariable cause-specific hazard regression)**	
Textured	ref
Smooth	0.96 (0.66, 1.41)
Polyurethane	**1.48 (1.08, 2.04)**
Adjusted (multivariable cause-specific hazard regression)*	
Textured	ref
Smooth	0.82 (0.45, 1.50)
Polyurethane	1.08 (0.51, 2.29)

Value in bold is statistically significant. HR_cs_, cause-specific hazard ratio. *Adjusted for acellular dermal matrix/mesh use, age, ASA classification, BMI, indication for reconstruction, institutional volume per year, laterality, number of applied infection control measures during implant insertion, plane, previous radiotherapy, smoking status, type of reconstruction, and taking clustering within patients into account. Incision site could not be included as a confounder because one of the categories contained zero events.

### Revision indications

Considering all surface-related revisions, the most frequently reported indications were asymmetry (27.4%), breast pain (26.6%), deep wound infection (26.4%), and capsular contracture (23.5%) (*Table S3*).

### Subgroup analyses

#### Capsular contracture

**Fig. 2:**
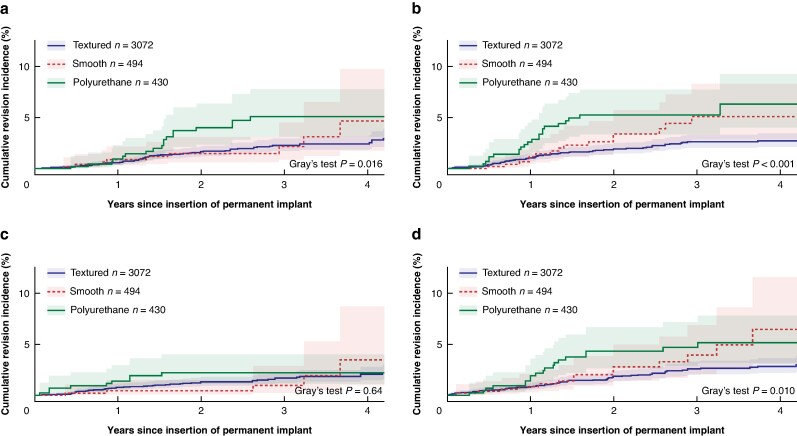
**a–d**. **Cumulative incidence of revision due to specific complications of permanent breast implants inserted for postmastectomy reconstruction, stratified according to implant surface** **a** Capsular contracture, **b** asymmetry, **c** implant malposition, and **d** breast pain. The curves have not been adjusted for confounders and clustering within patients has not been taken into account. Confidence intervals are displayed by the shadows of the curves. Numbers at risk are equal to *Fig. 1*.

Revision due to capsular contracture occurred in 2.0% (*n* = 62) of textured, 1.8% (*n* = 9) of smooth, and 4.4% (*n* = 19) of polyurethane implants. Over the entire follow-up period, the cumulative incidence of revision due to capsular contracture differed significantly between breast implant surface types (*P* = 0.016). At 4 years follow-up, no significant difference in cumulative incidence was observed between textured (2.4%; 95% c.i. = 1.8% to 3.1%), smooth (4.7%; 95% c.i. = 1.8% to 9.8%), and polyurethane-covered implants (5.1%; 95% c.i. = 3.1% to 7.8%) (*Fig. 2a*). In the multivariable cause-specific hazard regression, polyurethane implants showed an increased hazard of revision due to capsular contracture (HR_cs_ = 2.49; 95% c.i. = 1.24 to 5.01) compared to textured implants (*Table 3*).

**Table 3:** Cause-specific hazard regression of revision due to specific complications of permanent breast implants inserted for post-mastectomy reconstruction

	Capsular contracture	Asymmetry	Device malposition	Breast pain
	HR_cs_ (95% c.i.)	HR_cs_ (95% c.i.)	HR_cs_ (95% c.i.)	HR_cs_ (95% c.i.)
**Unadjusted (univariable cause-specific hazard regression)**				
Textured	ref	ref	ref	ref
Smooth	1.16 (0.53, 2.51)	1.66 (0.88, 3.10)	0.80 (0.31, 2.08)^†^	1.58 (0.82, 3.02)^†^
Polyurethane	**2.15 (1.20, 3.88)**	**2.56 (1.49, 4.41)**	1.35 (0.57, 3.16)^†^	**2.08 (1.20, 3.60)^†^**
Adjusted (multivariable cause-specific hazard regression)*				
Textured	ref	ref	ref	Ref
Smooth	0.93 (0.33, 2.65)	1.16 (0.56, 2.40)	0.72 (0.24, 2.14)^†^	1.37 (0.66, 2.85)^†^
Polyurethane	**2.49 (1.24, 5.01)**	**2.31 (1.33, 4.02)**	1.73 (0.75, 3.99)^†^	**2.35 (1.26, 4.38)^†^**

Values in bold are statistically significant. HR_cs_, cause-specific hazard ratio. *Adjusted for acellular dermal matrix/mesh use, age, ASA classification, BMI, indication for reconstruction, institutional volume per year, laterality, number of applied infection control measures during implant insertion, plane, previous radiotherapy, smoking status, type of reconstruction, and taking clustering within patients into account. †The proportional hazards assumption was violated for implant surface.

#### Asymmetry

Revision due to asymmetry occurred in 2.1% (*n* = 66) of textured, 3.0% (*n* = 15) of smooth, and 5.6% (*n* = 24) of polyurethane-covered implants. Over the entire follow-up period, the cumulative incidence of revision due to asymmetry differed significantly between breast implant surface types (*P* < 0.001). At 4 years follow-up, it differed significantly between polyurethane-covered (6.3%; 95% c.i. = 4.0% to 9.3%) and textured implants (2.7%; 95% c.i. = 2.1% to 3.5%). The cumulative incidence for smooth implants was equal to 5.1% (95% c.i. = 2.9 to 8.3) (*Fig. 2b*). In the multivariable cause-specific hazard regression, polyurethane-covered implants showed an increased hazard of revision due to asymmetry (HR_cs_ = 2.31; 95% c.i. = 1.33 to 4.02) compared to textured implants (*Table 3*).

#### Device malposition

Revision due to device malposition occurred in 1.5% (*n* = 47) of textured, 1.0% (*n* = 5) of smooth, and 2.1% (*n* = 9) of polyurethane-covered implants. Over the entire follow-up period, the cumulative incidence of revision due to device malposition did not differ significantly between breast implant surface types (*P* = 0.640). At 4 years follow-up, no significant difference in cumulative incidence was observed between textured (2.1%; 95% c.i. = 1.5% to 2.8%), smooth (3.5%; 95% c.i. = 1.0% to 8.7%), and polyurethane-covered implants (2.2%; 95% c.i. = 1.1% to 4.0%) (*Fig. 2c*). The proportional hazard assumption was violated for implant surface in the multivariable cause-specific hazard regression; however, there was no statistically significant difference in hazard ratios (*Table 3*).

#### Breast pain

Revision due to breast pain occurred in 2.2% (*n* = 68) of textured, 2.8% (*n* = 14) of smooth, and 4.7% (*n* = 20) of polyurethane-covered implants. Over the entire follow-up period, the cumulative incidence of revision due to breast pain differed significantly between breast implant surface types (*P* = 0.010). At 4 years follow-up, no significant difference in cumulative incidence was observed between textured (2.9%; 95% c.i. = 2.2 to 3.6), smooth (6.5%; 95% c.i. = 3.1 to 11.6), and polyurethane-covered implants (5.2%; 95% c.i. = 3.2 to 7.8) (*Fig. 2d*). In the multivariable cause-specific hazard regression, there was a statistically significant difference in hazards of revision due to breast pain between polyurethane-covered and textured implants. However, the proportional hazard assumption was violated for implant surface type (*Table 3*).

## Discussion

This study used prospective data from the Dutch Breast Implant Registry to compare cumulative incidences and hazards of surface-related revisions among textured, smooth, and polyurethane-covered implants after implant-based breast reconstruction. It is the first prospective, population-based study using real-world data from a nationwide registry to compare the performance of these implant types. Although 90.4% of breast implants required no surface-related revision, the cumulative incidence over the entire follow-up period differed significantly between implant surface types.

Polyurethane-covered implants showed a notable increase in cumulative surface-related revision incidence between 1 and 2 years’ follow-up, which levelled off thereafter. Consequently, by 4 years it did not differ significantly between textured (11.1%), smooth (13.0%), and polyurethane-covered implants (16.1%). Multivariable cause-specific hazard regression revealed no significant differences in surface-related revision between implant surface types. Subgroup analyses focusing on revisions for capsular contracture and asymmetry, however, showed significantly higher hazards for polyurethane-covered implants than for textured implants.

The cumulative incidences of revision in this study align with those reported by the Australian Breast Device Registry^14^. Comparative prospective trials investigating revisions by implant surface type remain limited. The largest prospective studies on outcomes following textured implants are manufacturer-initiated post-approval studies, which have reported revision rates of 18.0–31.8% at 3–5 years^53,54^. Prospective data on polyurethane-covered implants used for breast reconstruction are scarce, with one study reporting a 5% revision rate after 159 pre-pectoral IBBRs with a mean follow-up of 11 months^55^.

The relatively lower cumulative incidences of revision in the current study may be partially attributed to its specific focus on surface-related revisions and the low incidence of prior radiotherapy (8.6%). Radiotherapy is a known risk factor for increased revisions, as shown by population-based data from Canada, where 16.1% of immediate IBBRs received radiotherapy^56^. Dutch guidelines discourage immediate IBBR if adjuvant radiotherapy is indicated and recommend using autologous flaps following prior radiotherapy^7^.

Subgroup analysis revealed a higher cumulative incidence of revision for capsular contracture in polyurethane-covered implants (5.1%) compared to textured implants (2.4%) at 4 years. This was confirmed in the multivariable analysis. This contrasts with literature suggesting lower rates of capsular contracture for polyurethane-covered implants, with reported rates ranging from 1.8% to 8.1% after 4–9 years^24,25^. It should be noted that the current study only included capsular contractures that required revision surgery.

Only one retrospective study, with a median follow-up of 2.3 years, compared the capsular contracture rate of polyurethane-covered implants (8.1%) with textured implants (15.8%) after IBBR^26^. For textured implants, the present study observed a relatively low cumulative incidence of revision for capsular contracture of 2.4% compared to previously reported capsular contracture rates—with or without revision—of 5.9–17.2% after 3–5 years follow-up^28,53,54,57^.

Most previous studies lacked data on radiotherapy, a major risk factor for capsular contracture. Some studies suggested that polyurethane-covered implants mitigate the impact of radiotherapy compared to textured implants^25,26^. However, the literature on capsular contracture following polyurethane-covered IBBR is of limited quality due to inadequate study designs. In addition, conflicts of interest may have influenced some studies. Nonetheless, manufacturers confidently assert benefits of polyurethane in their marketing.

In both unadjusted and adjusted analyses, polyurethane-covered implants had a significantly higher chance of revision for asymmetry compared to textured implants. Previous studies have reported asymmetry rates of 11% for polyurethane-covered implants after 12 months, compared to 4.5–8.7% for textured implants over 3–5 years^53,54,58^. Asymmetry can result from various causes, including malposition, rotation, and capsular contracture.

A systematic review reported malposition rates of 2.1–4.0% at 6 years follow-up for textured implants compared to 1.8% for polyurethane-covered implants^24^. Note that these results originated from non-comparative studies with substantial design flaws. A prospective, multicentre, non-randomized trial showed a significantly lower rate of revision surgery for asymmetry after primary reconstruction with textured (3.9%) compared to smooth implants (11.1%) over 10 years^23^. It is important to highlight that smooth implants in the current study were predominantly round. Consequently, these reconstructions cannot develop asymmetry due to rotation, unlike reconstructions with anatomical textured implants.

Quality of life, costs, and cost-effectiveness remain key research areas in breast reconstruction comparing implant-based and autologous techniques. Quality-of-life differences are moderate and limited to specific scales in condition-specific questionnaires^6,59,60^. Complications after autologous reconstruction appear to negatively influence quality of life more than complications following IBBR^6,61^. Some have hypothesized that IBBR, over long-term follow-up, requires more revision surgeries than autologous reconstruction, leading to higher costs, lower quality of life, and reduced cost-effectiveness^62^. In contrast, autologous reconstruction primarily incurs expenses mainly during the initial surgery and short-term revisions, with more stable costs thereafter^62,63^. Long-term costs of autologous reconstruction were however still 21% higher than IBBR in a sensitivity analysis in which all IBBRs required reoperation after 10 years. IBBR has been demonstrated to be cost-effective compared to autologous reconstruction^63^. These comparisons are complex, and the outcomes of the present study add limited insight considering its medium-term follow-up.

The strengths of the present study include its prospective, nationwide design and independence of funding. The DBIR allows tracking implants using unique identifiers, even across different hospitals. Findings may have limited generalizability due to differences in healthcare systems, populations, guidelines, and surgical techniques. The multivariable analysis addressed confounders such as surgical techniques, prior radiotherapy, and laterality while accounting for clustering and competing risks. All breast reconstructions in this study were performed immediately following mastectomy, with exclusion of breasts with previous breast implant surgery, and without including reconstruction revisions, thus limiting population heterogeneity and potential selection bias. Despite these efforts, selection bias still may have influenced the results, in particular for polyurethane-covered implants, because earlier literature suggested that they may reduce the risk of capsular contracture, and surgeons may have thus preferentially choose these implants in patients perceived to be at higher risk.

Importantly, DBIR does not capture data on adjuvant radiotherapy following immediate reconstruction, which might have introduced confounding by indication, although none of the revised implants in this study were exposed to radiotherapy following original placement. Limitations also include a follow-up period of only 4 years and potential underreporting of revisions, particularly those involving implant repositioning without replacement. This may have led to an underestimation of the true chance of revision.

As the DBIR database and follow-up increase, future analyses may detect statistically significant differences that were not apparent in this study, as the incidence of revisions increases with time^29,30,32^. Observational studies like this are inherently susceptible to bias, requiring careful interpretation of results in the context of the complexities involved in decision-making regarding IBBR.

Future research should prioritize high-quality, prospective, comparative studies to assess complications and revision rates following IBBR. Registry-based research offers a valuable alternative to traditional randomized controlled trials^64^. Registries similar to DBIR are available in other countries and are well-suited for this type of research. A recent proof-of-concept study combining international registry data highlights the potential for generating high-quality evidence on breast implant safety^65^. Additionally, an exception to the General Data Protection Regulation allowing linking related registries, such as DBIR and the NABON Breast Cancer Audit, a comprehensive Dutch registry collecting essential data on breast cancer care, could greatly improve future research.

## Supplementary Material

znaf082_Supplementary_Data

## Data Availability

Data are not openly available, because they originate from a national registry (DBIR).
