# Supplementary material for: Comparison of revision surgery after implant-based breast reconstruction between smooth, textured, and polyurethane-covered implants: results from the Dutch Breast Implant Registry
Source: Br J Surg. 2025 May 17;112(5):znaf082. doi: 10.1093/bjs/znaf082 (PMC12084802; doi:10.1093/bjs/znaf082)
Supplement: znaf082_Supplementary_Data [file znaf082_supplementary_data.zip › Supplementary_Material.docx]

Comparison of Revision Surgery after Implant‐Based Breast Reconstruction Between Smooth, Textured and Polyurethane-Covered Implants: Results from the Dutch Breast Implant Registry

*Author list:*

J.X. Harmeling^1^, j.harmeling@erasmusmc.nl, MD

J. Juliët Vrolijk^2,3^, j.vrolijk@maastrichtuniversity.nl, MD

Erik Heeg^4^, heeg.erik@gmail.com, MD, PhD

Babette E. Becherer^1,5^, bebecherer@gmail.com, MD, PhD

Hinne A. Rakhorst^3,6^, rakhorst@gmail.com, MD, PhD

Eveline M.L. Corten^1^, e.corten@erasmusmc.nl, MD, PhD

Marta Fiocco^7,8,9^, m.fiocco@math.leidenuniv.nl, PhD

Marc A.M. Mureau^1,3^, m.mureau@erasmusmc.nl, MD, PhD

^1^ Department of Plastic and Reconstructive Surgery, Erasmus MC Cancer Institute, University Medical Center Rotterdam, Rotterdam, Zuid-Holland, The Netherlands

^2^ Department of Plastic and Reconstructive Surgery, Maastricht University Medical Center +, Maastricht, the Netherlands.

^3^ Dutch Institute for Clinical Auditing, Leiden, the Netherlands

^4^ Department of Plastic, Reconstructive and Hand Surgery, Amsterdam University Medical Centre, Amsterdam, The Netherlands

^5^ Allegro Medical, Hilversum, Noord-Holland, The Netherlands

^6^ Department of Plastic, Reconstructive, and Hand Surgery, Medisch Spectrum Twente, Enschede and ZGT Almelo, The Netherlands

^7^ Mathematical Institute, Leiden University, Leiden, The Netherlands

^8^ Department of Biomedical Data Science, Section Medical Statistics, Leiden University Medical Center, Leiden, The Netherlands

^9^ Princess Máxima Center for Pediatric Oncology, Utrecht, The Netherlands

*Corresponding author:*

Marc A.M. Mureau

Internal postal address Na-22.24

P.O. Box 2040

3000 CA Rotterdam

The Netherlands

m.mureau@erasmusmc.nl

Authors

**Supplementary Materials - Index**

| **Supplementary Figures and Tables** |  |
| --- | --- |
| Tables and figures | *page 3* |
|  |  |

**Supplementary Figures and Tables**

**Figure S1**. Flow chart of implant selection

**Table S1**. Classification of indications for revision surgery available in the Dutch Breast Implant Registry

**Table S2**. Univariable* cause-specific hazard regression of surface-related revision of permanent breast implants inserted for postmastectomy reconstruction

**Table S3**. Indications for surface-related revision per implant surface group

**Supplemental Digital Content 1.** DBIR Data Dictionary
