# Supplementary material for: Comparison of revision surgery after implant-based breast reconstruction between smooth, textured, and polyurethane-covered implants: results from the Dutch Breast Implant Registry
Source: Br J Surg. 2025 May 17;112(5):znaf082. doi: 10.1093/bjs/znaf082 (PMC12084802; doi:10.1093/bjs/znaf082)
Supplement: znaf082_Supplementary_Data [file znaf082_supplementary_data.zip › Table_S1.docx]

# **Table S1**. Classification of indications for revision surgery available in the Dutch Breast Implant Registry

| **Surface-related revision** | **Non-surface-related revision** |
| --- | --- |
| Asymmetry  Breast pain  Capsular contracture  Deep wound infection  Device malposition  Device rupture  Newly diagnosed breast cancer  Seroma or hematoma  Suspicion of or pathology-confirmed BIA-ALCL  Silicone extravasation  A recall  Breast implant-associated illness (BII) | Contralateral problem  Mastectomy flap problem  Patient dissatisfaction with volume  Patient request without health complaints  Skin necrosis or dehiscence  Skin scarring problems |
