# Supplementary material for: Comparison of revision surgery after implant-based breast reconstruction between smooth, textured, and polyurethane-covered implants: results from the Dutch Breast Implant Registry
Source: Br J Surg. 2025 May 17;112(5):znaf082. doi: 10.1093/bjs/znaf082 (PMC12084802; doi:10.1093/bjs/znaf082)
Supplement: znaf082_Supplementary_Data [file znaf082_supplementary_data.zip › Table_S2.docx]

# **Table S2**. Univariable* cause-specific hazard regression of surface-related revision of permanent breast implants inserted for postmastectomy reconstruction

| **Factor** | **HR_cs_ (95%CI)** |
| --- | --- |
| **Age in years** |  |
| <40 | 0.96 (0.70-1.32) |
| 40-49 | 1.20 (0.90-1.60) |
| 50-59 | ref |
| ≥60 | 1.20 (0.88-1.63) |
| **ASA classification** |  |
| I/II | ref |
| III+ | **1.91 (1.34-2.74)** |
| **BMI in kg/m^2^** |  |
| <25 | ref |
| ≥25 | 1.95 (0.75-1.20) |
| **Smoking status** |  |
| Not smoking | ref |
| Smoking | **1.52 (1.09-2.12)** |
| **Previous radiotherapy** |  |
| No | ref |
| Yes | **1.50 (1.08-2.09)** |
| **Institutional volume per year** |  |
| <75 | 1.03 (0.82-1.28) |
| ≥75 | ref |
| **Indication for reconstruction** |  |
| Therapeutic mastectomy | ref |
| Prophylactic mastectomy | 0.85 (0.63-1.15) |
| **Laterality** |  |
| Unilateral | ref |
| Bilateral | **0.79 (0.63-0.99)** |
| **Type of reconstruction** |  |
| Direct-to-implant | **0.61 (0.49-0.76)** |
| Two-stage | ref |
| **Unplanned TE revision** |  |
| No | ref |
| Yes | 1.31 (0.87-1.99) |
| Not applicable: direct-to-implant | **1.73 (1.36-2.19)** |
| **Incision site** |  |
| Nipple sparing | 1.00 (0.79-1.25) |
| Non-nipple sparing | ref |
| Other | 1.34 (0.77-2.32) |
| **Plane** |  |
| Total submuscular plane | 0.80 (0.63-1.02) |
| Other | ref |
| **Number of applied ICMs during implant insertion** |  |
| <4 | 0.81 (0.60-1.10) |
| 4 | 0.78 (0.61-1.00) |
| >4 | ref |
| **ADM/mesh** |  |
| No | ref |
| Yes | 1.40 (1.00-1.92) |
| **Implant shape** |  |
| Round | 1.01 (0.74-1.35) |
| Anatomical | ref |

*Abbreviations: ASA, American Society of Anaesthesiology; BMI, Body Mass Index; TE, Tissue Expander.
Age was divided into quartiles (cut-offs at percentiles 25, 50, and 75). For ASA classification, classes I and II were merged because they were considered clinically similar. For BMI, ≥25 kg/m^2^ was used as the cut-off point, because <25 kg/m2 was considered a healthy weight.
*Clustering of implants within patients has been taken into account.*
