# Supplementary material for: Comparison of revision surgery after implant-based breast reconstruction between smooth, textured, and polyurethane-covered implants: results from the Dutch Breast Implant Registry
Source: Br J Surg. 2025 May 17;112(5):znaf082. doi: 10.1093/bjs/znaf082 (PMC12084802; doi:10.1093/bjs/znaf082)
Supplement: znaf082_Supplementary_Data [file znaf082_supplementary_data.zip › Table_S3.docx]

# **Table S3**. Indications for surface-related revision per implant surface group

|  | Textured  (n events=285) | Smooth  (n events=38) | Polyurethane  (n events=60) |
| --- | --- | --- | --- |
| Asymmetry | 66 (23.2) | 15 (39.5) | 24 (40.0) |
| Breast pain | 68 (23.9) | 14 (36.8) | 20 (33.3) |
| Deep wound infection | 86 (30.2) | 8 (21.1) | 7 (11.7) |
| Capsular contracture | 62 (21.8) | 9 (23.7) | 19 (31.7) |
| Device malposition | 47 (16.5) | 5 (13.2) | 9 (15.0) |
| Seroma or hematoma | 45 (15.8) | 6 (15.8) | 1 (1.7) |
| Newly diagnosed breast cancer | 15 (5.3) | 1 (2.6) | 4 (6.7) |
| Breast implant-associated illness | 7 (2.5) | 1 (2.6) | 0 (0.0) |
| Implant rupture | 6 (2.1) | 0 (0.0) | 0 (0.0) |
| Suspicion of BIA-ALCL | 3 (1.1) | 0 (0.0) | 0 (0.0) |
| Silicone extravasation | 3 (1.1) | 0 (0.0) | 0 (0.0) |
| Recall | 1 (0.4) | 0 (0.0) | 0 (0.0) |
| PA-confirmed BIA-ALCL | 0 (0.0) | 0 (0.0) | 0 (0.0) |

*Abbreviations: BIA-ALCL, Breast Implant-Associated Anaplastic Large Cell Lymphoma; PA, pathology.
Values in parentheses are percentages. Multiple indications could be reported per revision procedure. Indications are listed from most frequently to least frequently reported for textured, smooth, and polyurethane implants combined.*
