# Supplementary figures and images for: Comparison of revision surgery after implant-based breast reconstruction between smooth, textured, and polyurethane-covered implants: results from the Dutch Breast Implant Registry
Source: Br J Surg. 2025 May 17;112(5):znaf082. doi: 10.1093/bjs/znaf082 (PMC12084802; doi:10.1093/bjs/znaf082)

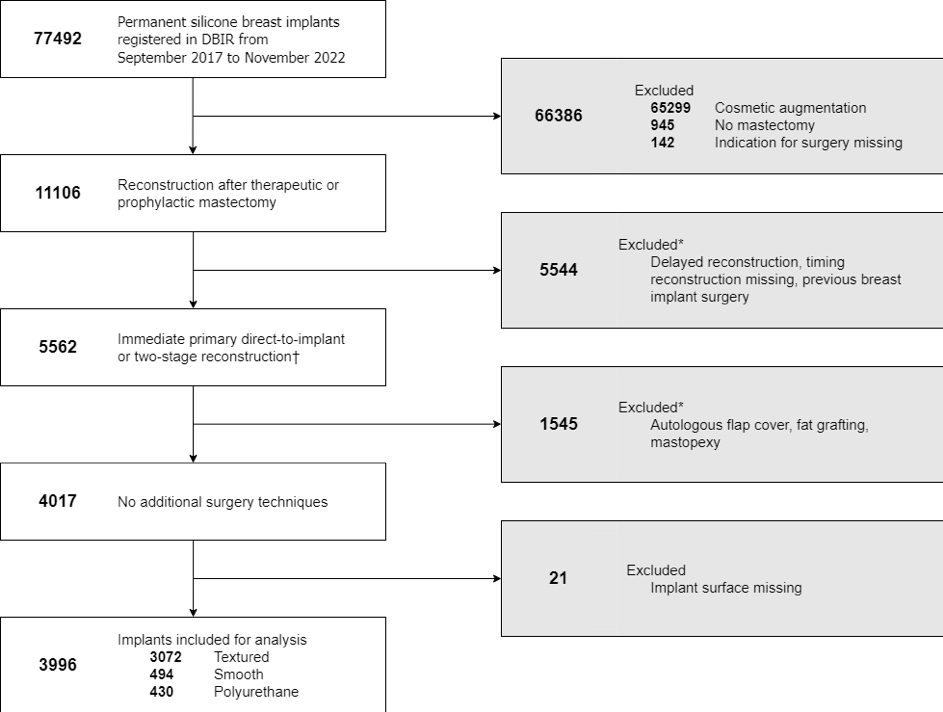

Supplement: znaf082_Supplementary_Data [file znaf082_supplementary_data.zip › Figure_S1.jpg]
